# Video Grading of Descemet Membrane Endothelial Keratoplasty Surgery to Identify Surgeon Risk Factors for Graft Detachment and Rebubbling: A Post Hoc Observational Analysis of the *Advanced Visualization In Corneal Surgery Evaluation* Trial

**DOI:** 10.1097/ICO.0000000000003181

**Published:** 2022-11-21

**Authors:** Marc B. Muijzer, Heleen Delbeke, Mor M. Dickman, Rudy M. M. A. Nuijts, Hanad Jimale, Chantal M. van Luijk, Saskia M. Imhof, Robert P. L. Wisse

**Affiliations:** * Utrecht Cornea Research Group, Ophthalmology Department, University Medical Center Utrecht, Utrecht, the Netherlands;; † Ophthalmology Department, University Hospital Leuven, Leuven, Belgium;; ‡ KU Leuven, Biomedical Sciences Group, Department of Neurosciences, Research group Ophthalmology; Leuven, Belgium; and; § University Eye Clinic, Department of Ophthalmology, Maastricht University Medical Center, Maastricht, the Netherlands.

**Keywords:** Descemet membrane endothelial keratoplasty, DMEK, video grading, graft detachment, rebubbling

## Abstract

The aim of this study was to explore video-graded intraoperative risk factors for graft detachment (GD) and rebubbling in Descemet membrane endothelial keratoplasty surgery.

**Methods:**

A post hoc analysis of 65 eyes of 65 pseudophakic subjects with Fuchs endothelial dystrophy that underwent Descemet membrane endothelial keratoplasty surgery as part of the *Advanced Visualization In Corneal Surgery Evaluation* trial. All surgical recordings were assessed by 2 graders using a structured assessment form. A multinominal regression was performed to estimate the independent effect of video-graded intraoperative factors on the incidence of GD and rebubbling. Secondary outcomes are corrected distance visual acuity and endothelial cell density.

**Results:**

In total, 33 GDs were recorded, of which 17 required rebubbling. No significant predictors for GD or rebubbling were identified. However, the results revealed 2 clinically relevant patterns. An unfavorable graft configuration (ie, wrinkled, tight scroll, or taco-shaped) and a gas-bubble size smaller than the graft diameter were associated with an increased risk of GD [odds ratio (OR) 2.5 and OR 2.26, respectively] and rebubbling (OR 2.0 and OR 2.60, respectively). Inversely, a larger gas-bubble size was associated with a reduced risk of GD (OR 0.37) and rebubbling (OR 0.36). At 3 and 6 months postoperatively, corrected distance visual acuity was poorer in subjects requiring a rebubbling and endothelial cell density loss was higher in subjects with a partial GD.

**Conclusions:**

Our analysis revealed that the gas-bubble size and graft shape/geometry seem to be relevant clinical factors for GD and rebubbling, whereas descemetorhexis difficulty, degree of graft manipulation, graft overlap, and surgical iridectomy were not associated with an increased risk.

## Abstract 2

Supplemental Digital Content is Available in the Text.

Descemet membrane endothelial keratoplasty (DMEK) is the preferred surgical procedure for symptomatic irreversible corneal endothelial dysfunction.^1,2^ Postoperative graft detachment (GD) is the most common complication, affecting about 1 in 5 patients.^2–6^ Detachments often require secondary surgery (ie rebubbling) that is burdensome for patients, may jeopardize graft survival, and strains health care resources.^7,8^

The underlying cause of GD is multifactorial. A wide range of risk factors have been proposed related to donor and patient characteristics and surgery.^8–13^ Reported intraoperative risk factors for GD and rebubbling include direct manipulation of the graft,^10,14–16^ use of viscoelastic,^17^ use of sulfur hexafluoride gas or air,^18,19^ insufficient graft support by the gas bubble in the anterior chamber,^19–24^ Descemet remnant, or overlap between donor and recipient Descemet membrane.^25,26^ Unfortunately, most studies have focused on a single or a few intraoperative events in their analysis. Only a handful of studies investigated the effect of surgical manipulation during graft unfolding or the effect of variations in intraoperative tissue handling on outcomes, although both are deemed important by surgeons.

In this study, we qualitatively and quantitatively analyzed surgical videos from the prospective *Advanced Visualization In Corneal Surgery Evaluation* (ADVISE) trial to identify risk factors for GD and rebubbling. The aim of this explorative study was to obtain a better understanding of the impact of surgical factors on the incidence of GD and rebubbling and subsequently offer insights to improve the safety of our surgeries.

## MATERIALS AND METHODS

This article is a post hoc observational analysis of surgical recordings to identify risk factors for GDs and rebubbling procedures of the prospective ADVISE trial, an international noninferiority single-blinded RCT to investigate the utility of intraoperative optical coherence tomography (iOCT) in DMEK surgery. All procedures were performed in accordance with the Declaration of Helsinki, local and national laws regarding research, European directives with respect to privacy, and 2010 CONSORT standards for reporting RCTs.^27^ The study was approved by the Ethics Review Boards in the Netherlands (Medical Ethics Committee Utrecht, file no. 18–487) and Belgium (Ethical committee Leuven, file no. S61527). The details of this trial are previously reported and registered at clinicaltrials.gov (no° NCT03763721).^28^

Subjects underwent surgery between December 2018 and April 2021 at the University Medical Center Utrecht (n = 39), University Hospital Leuven (n = 14), or Maastricht University Medical Center (n= 14). Inclusion criteria were pseudophakic adult patients with irreversible corneal endothelial dysfunction resulting from Fuchs endothelial corneal dystrophy, eligible for DMEK surgery. Subjects were excluded if they would undergo human leukocyte antigen-matched keratoplasty, underwent previous keratoplasty, were scheduled for combined phacoemulsification procedures, and had ocular comorbidities other than ocular surface disease, stable open-angle glaucoma, and mild age-related macular degeneration/changes. Only 1 eye per subject was enrolled.

Each patient underwent a comprehensive ophthalmic examination preoperatively and 1 day, 1 week, 1 month, 3 months, and 6 months after surgery. Here, we report the baseline, 3 months, and 6 months measurements in detail. The ophthalmic examination was previously reported.^28^ An optometrist measured the corrected distance visual acuity (CDVA) using an Early Treatment Diabetic Retinopathy Study letter chart at a distance of 4 m, and endothelial cell counts were assessed with specular microscopy (EM4000; Tomey, Nagoya, Japan, and SP-3000; Topcon, Nagoya, Japan).

### Surgical Procedure

Donor grafts were allocated by the Dutch Transplant Foundation (Nederlandse Transplantatie Stichting, Leiden, the Netherlands). The grafts were cultured and provided prepeeled by the ETB-Bislife (Beverwijk, the Netherlands), with a minimum endothelial cell density (ECD) of 2300 cells/mm^2^ and a diameter of 8.5 mm. No graft markings were used. Before surgery, 27 subjects underwent Nd:YAG laser iridotomy at the 6-o'clock position. In the other 38 subjects, surgical iridectomy was performed using a 27-gauge needle and Price hook at the 6-o'clock position after the descemetorhexis during surgery.

All surgical procedures were performed by experienced corneal surgeons (H.D., R.M.M.A.N, M.M.D., and R.P.L.W.). The surgical procedure was standardized because all included cases were part of the ADVISE trial. The study protocol has been reported in detail before; both arms of the trial were combined in this report on video grading.^28^ In short, the procedure consisted of a 9-mm descemetorhexis, and subsequently, the graft was stained and inserted in the anterior chamber using a glass injector through a 2.8-mm incision. A no-touch technique was used to unfold and position the graft. As per randomization of the ADVISE trial, iOCT was not available in half of the cases (reference; n = 33) and the intraocular pressure was raised above physiological limits (ie, overpressure) for 8 minutes at the end of surgery. In the other half of the cases (iOCT-optimized protocol intervention; n = 32), a brief AC fill was performed to adhere the graft and the iOCT was used to check for complete adherence of the graft without overpressurizing the eye. In all cases, air was replaced by sulfur hexafluoride 20% gas, and the size of the gas bubble was reduced to cover the graft (ie, the same size as the graft). After surgery, patients remained strictly supine for 2 hours at the hospital and were instructed to remain supine for the following 24 hours.

### Video Analysis

**FIGURE 1.**
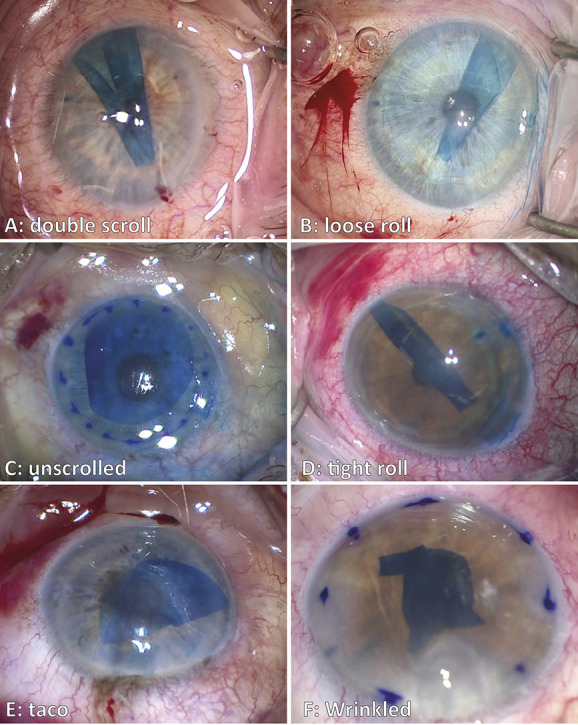
Coding the various graft shapes directly after insertion in the AC, based on graft geometry and number of folds. The distinctive graft shapes were grouped as either favorable (A, B, C) or unfavorable (D, E, F).

All surgical video recordings were analyzed by 2 graders (M.B.M. and H.J.) using a standardized assessment form. One grader was masked regarding the clinical outcomes of the surgery. The overall skin-to-skin surgical time and duration of surgical steps (eg, descemetorhexis, graft unfolding, etc.) were meticulously recorded. The difficulty of the descemetorhexis was coded in 3 groups based on time (ie, fast: ≤5 minutes, average: >5–≤10 minutes, and slow: >10 minutes). Directly after insertion of the graft into the AC, graft configuration was determined based on geometry and number of folds. As such, 6 distinct shapes were distinguished: double scroll, tight roll, loose roll, taco, unscrolled, and wrinkled (Fig. 1). The assessment of the surgeon and observations from the surgical video were cross-checked to identify agreement. Based on the incidence of GD/rebubbling and expert opinion, the shapes were considered favorable and unfavorable to unfold and position during surgery. The shapes *double scroll, loose roll,* and *unscrolled* were coded favorable graft shapes to unfold, whereas the shapes *tight roll, taco,* and *wrinkled* were coded unfavorable. Ease of graft unfolding was classified in 4 mutually exclusive ordinal groups depending on the required manipulation and time to unfold/position the graft, as described by Maier et al.^14^ All manual manipulations of the graft (eg, bubble bumping, positioning with forceps, and pushing in place with a cannula) were recorded and coded in 3 nominal groups: the category *external manipulation* indicates only corneal swiping/tapping was used to unfold the graft, regardless of the time spent. *Indirect internal manipulation* refers to bubble bumping or fluid–air exchange to unfold the graft in addition to external manipulation. *Direct internal manipulation* was scored if the graft was manually unfolded or positioned using surgical instruments in addition to external and *indirect internal manipulation*. After fixation of the graft with gas, the size of the gas bubble was assessed, compared with the graft size, and coded in 3 nominal groups: a gas-bubble size *equal* to the graft diameter, *larger* than the graft diameter, and *smaller* than the graft diameter. Centering of the graft was determined in relationship to the descemetorhexis area and coded dichotomously; a graft was considered centered if it was completely within the descemetorhexis and decentered if the graft overlapped with the descemetorhexis edge.

### Statistical Analysis

The outcome variables of this study are the incidence of GDs and rebubbling procedures. A GD was defined as any nonadherence of the graft noticeable on slit-lamp examination and AS-OCT imaging (Utrecht and Leuven: Zeiss Cirrus 5000, Zeiss Meditec, Oberkochen, Germany; Maastricht: Casia SS-1000, Tomey, Nagoya, Japan) at any time point within 3 months after surgery.^29^ A rebubbling was defined as the reinjection of gas under the graft after a GD. A rebubbling was performed if the graft was >30% detached or involved the visual axis. A graft failure was defined as a nonclearing of the cornea, an increase of corneal edema between the 6 and 3 months visit, or cases with repeated GD requiring rebubbling (>2 times). Secondary outcomes are postoperative ECD and CDVA. The Early Treatment Diabetic Retinopathy Study letter score of the CDVA was converted to logarithm of the minimum angle of resolution units by multiplying the number of letters read by −0.02 log units and adding 1.7 log units.^30^

Missing observations of CDVA and ECD were considered missing at random and imputed using multiple imputation. Missing measurements of subjects who developed a graft failure were considered missing not at random and not imputed. The number of imputations was equal to the maximum percentage of missing data plus 1. Two surgical recordings were missing, and the results were not imputed.

Here, all cases were video analyzed irrespective of the randomization in the ADVISE trial. Consequences of the randomization (ie, prolonged overpressure of the eye) were entered as factors in the multivariable model. Subjects were post hoc assigned in 3 groups: completely attached graft, GD that did not require rebubbling, and GD that required rebubbling. Group differences were analyzed using a one-way ANOVA or Kruskal–Wallis test, as appropriate. Correction for multiple comparisons was performed using the Bonferroni correction. A 2-sided *P* value <0.05 was considered statistically significant. Internal consistency of the video grading between the 2 graders was assessed using the Cohen kappa.^31^

A multivariate multinomial regression analysis was performed to analyze the independent effect of intraoperative factors on the incidence of GD and rebubbling. Predictors included graft manipulation, graft shape, graft centering, gas-bubble size, method of peripheral iridectomy (laser vs. surgical), and overpressure duration. The regression analysis was adjusted for center, to account for differences between surgeons and number of surgeries, and donor age because this may influence scroll tightness.^32^ The analysis was not adjusted for randomization used in the trial because no differences between the treatment arms regarding graft unfolding and manipulation were observed in the primary analysis of the trial. All statistical analyses were performed using R statistical software version 4.0.3 (Comprehensive R Archive Network, Vienna, Austria).

## RESULTS

A total of 65 eyes of 65 patients were included for analysis. In total, 33 (51%) GDs were recorded over the study period, of which 17 (26%) required rebubbling. Of these 33 detachments, 3 cases underwent retransplantation for primary graft failure, and all 3 cases were preceded by a rebubbling. The average time between surgery and detection of detachment measured 7.54 days (SD: ±9.44, range: 1–32 days), and the average detached area measured 31% of the surface of the graft (range: 8%–100%) as previously reported.^29^ One repeated rebubbling was performed during the course of the study. No significant differences regarding the incidence of adverse events were found between the treatment arms nor between centers. Additional details of the ADVISE trial are previously reported, including the rate of adverse events between treatment arms and centers.^28^

The internal consistency between the 2 graders (M.M. and H.J.) regarding video grading was considered strong (Cohen kappa: 0.84 ± 0.17; agreement: 90%).^31^ Baseline patient and donor characteristics are displayed in Table 1. At baseline, statistically significant differences were found between the groups for patient age, CDVA, and donor ECD.

**TABLE 1.** Baseline Patient and Donor Characteristics Stratified in Three Post Hoc Groups Based on Postoperative Treatment Success

	Graft Attached (n = 32)	Graft Detached (n = 33)		*P*
		No Rebubbling (n = 16)	Rebubbling (n = 17)	
Recipient characteristics				
Sex (female), n (%)	16 (50.0)	8 (50.0)	10 (58.8)	0.822*
Age (yr), mean (SD)	72 (6.97)	72 (5.48)	76 (5.27)	0.040§
CDVA (logMAR), mean (SD)	0.37 (0.23)	0.57 (0.32)	0.37 (0.15)	0.021§
Pachymetry (μm), mean (SD)	608 (61.47)	669 (71.36)	600 (151.17)	0.080§
Corneal edema present, n (%)	11 (34.4)	9 (56.2)	8 (47.1)	0.328*
Descemet folds present, n (%)	2 (6.2)	2 (12.5)	4 (23.5)	0.215*
Bullae present, n (%)	4 (12.5)	4 (25.0)	1 (5.9)	0.270*
Laser iridotomy, n (%)	11 (34.4)	10 (62.5)	6 (35.3)	0.146*
Donor characteristics				
Age (yr), mean (SD)	72 (4.97)	76 (5.72)	75 (5.45)	0.101§
ECD (cells/mm^2^), mean (SD)	2763 (193)	2633 (150)	2688 (136)	0.049§

None of the P-values were significant after correction for multiple comparison using the Bonferroni method.

* Kruskal–Wallis test.

† One-way ANOVA.

logMAR, logarithm of the minimum angle of resolution.

### Video-Grading Outcomes

The various categories of tissue handling and surgical manipulation did not show significant differences between the 3 post hoc groups. In other words, intraoperative factors of detached grafts and detached grafts requiring rebubbling on average did not differ from uneventful postoperative courses (Table 2). It should be noted that this study was not powered on video-graded surgical manipulations, and *P* values contribute little additional value. Although at first glance, analysis of the videos revealed considerable relative differences between the groups regarding the gas-bubble size at the end of surgery and postoperative adverse events, a smaller bubble was more prevalent in cases which subsequently developed a detachment (attached: 10%; all detachments: 21%). Inversely, a gas-bubble size greater than the graft diameter was more prevalent in completely attached grafts (attached: 32%; all detachments: 15%). The prevalence of an unfavorable graft shape (ie, *wrinkled, tight scroll, or taco-shaped)* was higher in cases that developed a GD (attached: 23%; all detachments: 44%).

**TABLE 2.** Overview Video Analysis Assessment

	Graft Attached (n = 32)	Graft Detachment (n = 33)		*P*
		No Rebubbling (n = 16)	Rebubbling (n = 17)	
Descemetorhexis difficulty grade, n (%)				0.946*
Fast (≤5 minutes)	20 (64.5)	10 (71.4)	10 (58.8)	
Average (>5–≤10 minutes)	7 (22.6)	3 (21.4)	5 (29.4)	
Slow (>10 minutes)	4 (12.9)	1 (7.1)	2 (11.8)	
Descemet remnants present,§ n (%)	1 (3.2)	0 (0.0)	0 (0.0)	0.602*
Graft shape,‖ n (%)				0.130*
Unfavorable shapes	7 (22)	7 (43.8)	8 (47)	
Tight roll	1 (3.1)	3 (18.8)	0 (0.0)	
Taco	4 (12.5)	1 (6.2)	4 (23.5)	
Wrinkled	2 (6.2)	3 (18.8)	4 (23.5)	
Favorable shapes	24 (75.4)	9 (56.2)	9 (53)	
Double scroll	11 (34.4)	7 (43.8)	3 (17.6)	
Loose roll	9 (28.1)	2 (12.5)	4 (23.5)	
Unscrolled	4 (12.9)	0 (0.0)	2 (11.8)	
Graft unfolding grade¶#, n (%)				0.736*
Grade 1	6 (19.4)	2 (12.5)	3 (17.6)	
Grade 2	11 (35.5)	8 (50.0)	9 (52.9)	
Grade 3	3 (9.7)	2 (12.5)	0 (0.0)	
Grade 4	11 (35.5)	4 (25.0)	5 (29.4)	
Graft manipulations,§ n (%)				0.443*
External manipulation only	7 (22.6)	1 (6.2)	4 (23.5)	
Indirect internal manipulation	13 (41.9)	11 (68.8)	8 (47.1)	
Direct internal manipulation	11 (35.5)	4 (25.0)	5 (29.4)	
Graft centering,# n (%)				0.577*
Decentered	14 (46.7)	8 (53.3)	6 (35.3)	
Gas-bubble size,§ n (%)				0.452*
Equal to graft diameter	18 (58.1)	9 (60.0)	11 (64.7)	
Smaller than graft diameter	3 (9.7)	3 (20.0)	4 (23.5)	
Larger than graft diameter	10 (32.3)	3 (20.0)	2 (11.8)	
Surgical duration (minutes)§ mean (SD)				
Surgical skin-to-skin time	34.16 (10.23)	34.53 (8.59)	37.65 (13.38)	0.454**
Descemetorhexis duration	5.39 (4.92)	4.37 (3.10)	5.21 (5.35)	0.637**
Graft unfolding duration	4.44 (4.01)	6.10 (7.66)	6.67 (10.02)	0.257**

None of the P-values were significant after correction for multiple comparison using the Bonferroni method.

* Kruskal–Wallis test between attached grafts, detached grafts not requiring rebubbling, and detached grafts requiring rebubbling.

§ Two missing values.

‖ Three missing values.

¶ Grade I: graft lamella primarily oriented correctly in the anterior chamber, straight and direct unfolding and centering; grade II: slightly complicated, indirect unfolding and centering (duration less than 5 min); grade III: difficult indirect unfolding and centering (duration longer than 5 min), repeated air injection with BSS exchange necessary; and grade IV: direct manipulation of the graft lamella for unfolding and centering by a cannula or a pair of forceps.

# One missing value.

# Decentered was defined as the graft overlapping with recipient Descemet membrane.

** One-way ANOVA between attached grafts, detached grafts not requiring rebubbling, and detached grafts requiring rebubbling.

**FIGURE 2.**
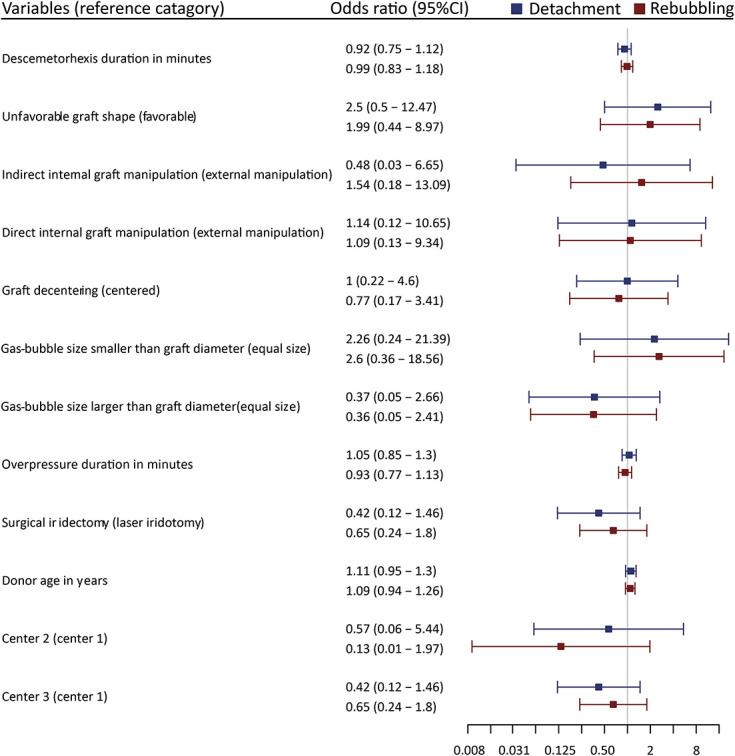
OR and 95% confidence interval of video-graded intraoperative factors on the incidence of a GD and rebubbling compared with cases with a fully attached graft. Per parameter, the reference category is noted between paracenteses. The analysis was adjusted for donor age and center.

To investigate independent effects and identify potential patterns that predict treatment outcomes, we conceptualized a multinomial regression model including surgical risk factors and confounding factors. No statistically significant predictors were identified in the analysis, although again the same 2 clinically relevant trends were identified: gas-bubble size at the end of surgery and graft shape after insertion (Fig. 2), Supplemental Digital Content (see Supplemental Table 1, http://links.lww.com/ICO/B459). A larger gas bubble at the end of surgery had a lower risk of GD and rebubbling [odds ratio (OR): 0.37 and 0.36, respectively]. Inversely, a smaller gas bubble had an increased risk of GD and rebubbling (OR: 2.26 and 2.60, respectively). Second, an unfavorable graft shape was associated with an increased risk of GD and rebubbling (OR: 2.50 and 1.99, respectively), independent of donor age. Other interesting outcomes are that surgical iridectomy was not related to an increased risk of GD and rebubbling (OR: 0.42 and 0.65, respectively). In decentered grafts, there is an apparent overlap between graft and host Descemet–endothelium, by some considered a risk factor for GDs.^26^ Our analysis did not show a consistent effect of graft decentering on the GD and rebubbling (OR: 1 and 0.77). Direct internal graft manipulation indicated a marginal increased risk of GD and rebubbling (OR: 1.14 and 1.09). Direct internal manipulation can be considered an iatrogenic trauma to the graft, which by some is considered related to unfavorable surgical outcomes, although a relation between ECD loss and internal manipulation was not found (data not shown).^10,14,16^

### Clinical Outcomes

At 3 and 6 months postoperatively, a significant difference was observed regarding the ECD in cases with a GD (3 months: *P* = 0.007; 6 months: *P* = 0.001) compared with cases with a completely attached graft and the detached grafts requiring a rebubbling. After adjustment for multiple comparisons and correction for baseline donor ECD, the difference between the groups was not significant. Furthermore, at 3 and 6 months postoperatively, subjects who underwent a rebubbling achieved a poorer CDVA compared with the other 2 groups, although this difference was not statistically significant after adjustment for multiple comparisons. A complete overview of postoperative clinical outcomes is shown in Table 3.

**TABLE 3.** Clinical Outcomes 3 and 6 Months After Surgery Stratified in Three Post Hoc Groups Based on Postoperative Treatment Success

	Graft Attached (n = 32)	Graft Detached (n = 33)		*P* †
		No Rebubbling (n = 16)	Rebubbling (n = 17)	
CDVA (logMAR), mean (SD)				
3 mo	0.13 (0.17)	0.13 (0.10)	0.25 (0.17)	0.033
6 mo	0.13 (0.21)	0.12 (0.15)	0.31 (0.27)	0.015
Pachymetry (μm), mean (SD)				
3 mo	473 (40.14)	492 (50.08)	461 (50.53)	0.160
6 mo	481 (49.27)	488 (60.31)	496 (56.06)	0.642
ECD (cells/mm^2^), mean (SD)				
3 mo	1948 (351)	1594 (379)	1717 (399)	0.007
6 mo	1920 (379)	1454 (414)	1804 (360)	0.001*
ECD loss‡ (cells/mm^2^), mean (SD)				
3 mo	814 (357)	1033 (364)	962 (451)	0.160
6 mo	842 (372)	1190 (419)	875 (356)	0.016

* *P* value significant at α ≤0.05 after correction for multiple comparison using the Bonferroni method.

† One-way ANOVA between attached grafts, detached grafts not requiring rebubbling, and detached grafts requiring rebubbling.

‡ Calculated as the difference between the specular microscopy measurement and the graft ECD.

logMAR, logarithm of the minimum angle of resolution.

## DISCUSSION

We report an analysis of surgical video recordings to explore risk factors for GD and rebubbling after DMEK. This study provides a rare opportunity to analyze surgical DMEK videos in-depth, in a well-controlled cohort of corneal transplant procedures performed by various surgeons in 3 clinics, with well-defined procedural trial parameters. We focused on the contribution of surgical manipulations, tissue handling, and (unnoticed) practice pattern variations to identify risk factors for GD and rebubbling. GDs have a notorious multifactorial origin, and several strategies to investigate this are reported, for example, case–control,^10^ case series,^9,13,25,33^ cohort studies,^11,14^ and registry studies.^8,34^ The added value of this study is the focus on the surgical course including clinical variations and surgeons handling, which enables further hypothesizing of the causality of these dreaded events.

The main findings of this study are that direct manipulation of the graft (ie, judicious grabbing with a pair of forceps) is not associated with poor surgical outcome nor was overlap of the graft with host Descemet membrane. The gas-bubble size at the end of surgery did seem clinically relevant: bigger is better in maintaining an attached graft. Commensurate to the primary outcomes of the ADVISE trial,^28^ the length of overpressuring did not relate to the incidence of GDs or rebubbling procedures. Another relevant factor in our model was graft configuration, a tissue characteristic that cannot be influenced by the surgeon; an unfavorable graft shape was associated with an increased risk of GD and rebubbling. One could hypothesize that the graft shape is a proxy for overall graft unfolding difficulty, including the associated intraoperative challenges. Still, we found no correlation between the coding of graft shape and the metrics of graft unfolding difficulty (χ^2^ = 4.87, *P* = 0.18) and duration (χ^2^ = 62, *P* = 0.44) as suggested by Quilendrino et al and Maier et al.^14,33^ Apparently, there is still an unexplained variation in our statistical model that predicts postoperative adverse events.

There is limited evidence regarding the causality of surgical decision making and detachment/rebubbling rates. Our results underlines that it is very difficult to predict GD or rebubbling, based on how the surgery faired. Several recommended practices were not supported by our results, such as not directly manipulating the graft and preventing overlap with host Descemet membrane. One of those recommended practices is avoidance of direct manual manipulation of the graft, which may lead to endothelial damage and GD.^13,15,16,33,35–37^ However, the causality between direct manual manipulation and detachment of the graft is unclear and evidence limited. Maier et al and Leon et al reported that manual manipulation was associated with a higher incidence of GD, although no significant associations were found.^10,14^ In our study, we did not find a higher incidence of adverse events in cases with direct graft manipulation, rather it seemed to have a reduced risk. The direct tissue effects of direct manual manipulation on ECD were not investigated in this study, only the effects on clinically relevant end points of graft adherence.

In our study, we did not find an association between graft overlap with recipient Descemet membrane and GD or rebubbling contradicting the findings of Rock et al ^12^ and Tourtas et al.^25^ Furthermore, Muller et al reported that incomplete removal of Descemet membrane (ie, overlap with the recipient anterior banded layer) and ultrastructural changes were related to GD. However, they reported that overlap with full-thickness Descemet membrane did not result in GD on histological images.^26^ In our study, we did not account for the extent of overlap, and actual complete removal of Descemet membrane layers or other ultrastructural changes were not investigated.

On the other hand, our results support that a larger gas-bubble size may be protective for a GD as previously reported by Leon et al and Cirkovic et al.^10,20^ Leon et al^10^ found that an air fill <75% of the anterior chamber height was associated with an increased risk of GD (OR: 2.66; *P* = 0.027). Similarly, Cirkovic et al^20^ reported that an 80% fill of the anterior chamber was significantly associated with a decreased incidence of rebubbling (*P* = 0.032). Pralits et al^21^ showed that graft support is dependent on the gas-bubble coverage of the graft. They demonstrated that a 63% fill already leads to incomplete coverage of the graft in different gaze directions independent of the type of gas filling. A larger gas bubble may mitigate the decrease of air-bubble size by leakage and half-life time of the tamponade agent.^38^

Endothelial decay at 3 months and 6 months was higher in eyes with a partial GD compared with eyes with an attached graft and eyes that required a rebubbling. A similar association between partial detachments and endothelial cell loss was found by Baydoun et al^39^ (mean difference: 330 cells/mm^2^; 95% confidence interval: 208–452; *P* < 0.001). This may indicate that a partial GD compromises long-term graft viability and could form an argument for early rebubbling. Mechanical loss of endothelial cells as result of tissue manipulation during surgery seems unlikely as this did not differ from the other groups in our analysis. As a result, we can only speculate on the cause of this endothelial cell loss, which may be the result of a larger area to be repopulated or unrecorded mechanical causes or trauma inhibiting cellular processes.

Furthermore, several limitations should be addressed. This study was a post hoc analysis of a trial that was not powered to determine associations between intraoperative factors and GD/rebubbling. Notwithstanding, the data were derived from a well-controlled sample of corneal transplant procedures and the video analysis enables an objective in-depth observation of the surgical course. In this study, we focused on intraoperative factors affecting graft disadherence. However, an analysis including recipient and additional donor factors may reveal additional insights, including graft quality or posterior stromal scarring. Several factors were assessed in the video analysis (eg, presence of Descemet remnants and stromal damage resulting from the descemetorhexis), although not included in the analysis because the incidence of these factors either was low or could not reliably be estimated in the video analysis. Similarly, some factors that were scored are inherently subjective, for example, the surgeons' assessment of how loose/tight the DMEK rolls were. What is considered tight or loose DMEK roll by 1 surgeon can be different for another surgeon. In this study, we cross-checked the video feed with the surgeons assessment; however, as this process is subjective, we chose not to report on the scroll tightness. During determining the graft shape, grafts were only classified as a tight roll if the graders' assessment on the video image matched the surgeons' assessment. Information on noncompliance of the patient and resorption time of the gas bubble was not collected and could not retrospectively be retrieved.

In conclusion, using a structured video analysis, we explored intraoperative determinants for GD and rebubbling after DMEK. Our analysis revealed that the gas-bubble size and graft shape/geometry seem to be relevant clinical factors. GD and rebubbling were not associated with the degree of graft manipulation, graft positioning, surgical iridectomy, or overpressuring the eye.

## Supplementary Material
