# Supplementary material for: Video Grading of Descemet Membrane Endothelial Keratoplasty Surgery to Identify Surgeon Risk Factors for Graft Detachment and Rebubbling: A Post Hoc Observational Analysis of the Advanced Visualization In Corneal Surgery Evaluation Trial
Source: Cornea. 2022 Nov 21;42(9):1074–82. doi: 10.1097/ICO.0000000000003181 (PMC10392889; doi:10.1097/ICO.0000000000003181)
Supplement: Supplementary file 1 [file cornea-42-1074-s001.docx]

Supplementary table 1. Multinomial regression analysis between surgical factors and graft detachment.

|  | **Detachment, no rebubbling** | | **Detachment, rebubbling** | |
| --- | --- | --- | --- | --- |
| **Variables** | **OR (95%CI)** | **P-value** | **OR (95%CI)** | **P-value** |
| Descemetorhexis duration (minutes) | 0.915 (0.749-1.119) | 0.388 | 0.988 (0.831-1.176) | 0.895 |
| Graft shape: unfavorable (reference: favorable) | 2.501 (0.502-12.467) | 0.263 | 1.99 (0.441-8.973) | 0.371 |
| Graft manipulations: external (reference) | - | - | - | - |
| Graft manipulations: indirect | 0.478 (0.034-6.655) | 0.583 | 1.536 (0.18-13.086) | 0.694 |
| Graft manipulations: direct | 1.139 (0.122-10.655) | 0.909 | 1.091 (0.127-9.335) | 0.937 |
| Graft centering: decentered (reference: centered) | 0.995 (0.215-4.593) | 0.995 | 0.771 (0.174-3.408) | 0.731 |
| Gas bubble size: equal to graft diameter (reference) | - | - | - | - |
| Gas bubble size: smaller than graft diameter | 2.262 (0.239-21.398) | 0.477 | 2.602 (0.365-18.564) | 0.34 |
| Gas bubble size: larger than graft diameter | 0.368 (0.051-2.663) | 0.322 | 0.359 (0.053-2.408) | 0.291 |
| Overpressure duration (minutes) | 1.054 (0.851-1.305) | 0.631 | 0.932 (0.767-1.133) | 0.479 |
| Surgical iridectomy (reference: laser iridotomy) | 0.42 (0.12-1.463) | 0.173 | 0.653 (0.236-1.801) | 0.41 |
| Donor age (years) | 1.108 (0.946-1.298) | 0.202 | 1.087 (0.94-1.256) | 0.259 |
| Center 1 (reference) | - | - | - | - |
| Center 2 | 0.566 (0.059-5.439) | 0.622 | 0.134 (0.009-1.974) | 0.143 |
| Center 3 | 0.42 (0.12-1.463) | 0.173 | 0.653 (0.236-1.801) | 0.41 |
